# A Genome-Wide Identification and Expression Analysis of the Casparian Strip Membrane Domain Protein-like Gene Family in *Pogostemon cablin* in Response to p-HBA-Induced Continuous Cropping Obstacles

**DOI:** 10.3390/plants12223901

**Published:** 2023-11-19

**Authors:** Yating Su, Muhammad Zeeshan Ul Haq, Xiaofeng Liu, Yang Li, Jing Yu, Dongmei Yang, Yougen Wu, Ya Liu

**Affiliations:** 1 School of Breeding and Multiplication (Sanya Institute of Breeding and Multiplication), Hainan University, Sanya 572025, China; 2 School of Tropical Agriculture and Forestry, Hainan University, Danzhou 571737, China

**Keywords:** *Pogostemon cablin*, *CASPL* family, bioinformatics, Casparian strip, expression analysis

## Abstract

Casparian strip membrane domain protein-like (*CASPL*) genes are key genes for the formation and regulation of the Casparian strip and play an important role in plant abiotic stress. However, little research has focused on the members, characteristics, and biological functions of the patchouli *PatCASPL* gene family. In this study, 156 *PatCASPL* genes were identified at the whole-genome level. Subcellular localization predicted that 75.6% of *PatCASPL* proteins reside on the cell membrane. A phylogenetic analysis categorized *PatCASPL* genes into five subclusters alongside *Arabidopsis CASPL* genes. In a cis-acting element analysis, a total of 16 different cis-elements were identified, among which the photo-responsive element was the most common in the *CASPL* gene family. A transcriptome analysis showed that p-hydroxybenzoic acid, an allelopathic autotoxic substance, affected the expression pattern of *PatCASPLs*, including a total of 27 upregulated genes and 30 down-regulated genes, suggesting that these *PatCASPLs* may play an important role in the regulation of patchouli continuous cropping obstacles by affecting the formation and integrity of Casparian strip bands. These results provided a theoretical basis for exploring and verifying the function of the patchouli *PatCASPL* gene family and its role in continuous cropping obstacles.

## 1. Introduction

*Pogostemon cablin* (Blanco) Benth is a perennial herb or semi-shrub plant of Labiatae, mainly distributed in the tropical and subtropical regions of Asia [1], such as India, Sri Lanka, Malaysia, Indonesia, and the Philippines [2]. In China, *P. cablin*, also called ‘Guanghuoxiang’, is mainly distributed in Hainan and Guangdong provinces [3,4]. According to the cultivation and production areas, it can be divided into four cultivation types, ‘Hainan Guanghuoxiang’ (Nanxiang), ‘Zhanjiang Guanghuoxiang’ (Zhanxiang), ‘Shipai Guanghuoxiang’ (Paixiang), and ‘Zhaoqing Guanghuoxiang’ (Zhaoxiang) [5,6]. As one of the ‘Ten Southern Medicines’ and a traditional Chinese medicine, it has been used for aromatic dampness, clearing heat, and as an antiemetic [7,8], and shows important medicinal and economic value [9]. However, continuous cropping obstacles are a key problem in the cultivation and production of *P. cablin*, which seriously affects its yield and quality [10,11]. Previous studies have found that the deterioration of soil physiochemical properties, the accumulation of allelochemicals, and the imbalance of microbial communities are the main causes of continuous cropping obstacles [12,13,14]. Among them, p-hydroxybenzoic acid (p-HBA) has been proved as a key allelochemical inducing the occurrence of *P. cablin* continuous cropping obstacles [15,16]. However, how does p-HBA induce the occurrence of *P. cablin* continuous cropping obstacles? Which signaling pathways are involved in the regulation process? These issues need to be further studied.

The Casparian strip is a bolted and lignified band-thickening wall structure surrounded by the radial wall and transverse wall of the endothelial cells, which plays an important role in screening, blocking, and cutting off unwanted ions or macromolecules into the vascular column [17,18]. It has been found that the formation and regulation of the Casparian strip involve multiple genes and signaling pathways [19,20]. It mainly contains the following key genes: Casparian strip membrane domain proteins (CASPs) [21], leucine receptor kinase (GSO1/SGN3) [22], enhanced suberin 1 (ESB1) [23], MYB domain protein 36 (MYB36) [24], and endodermis Casparian strip integrity factors 1/2 (CIF1/2) [25]. Among them, *CASPLs* are pivotal membrane proteins specifically expressed in the Casparian strip formation region. These proteins contain a four-transmembrane domain with cytoplasmic amino and carboxyl ends and conserved extracellular loops, which play a key role in the formation of the Casparian strip [26,27]. At present, the number of *CASPL* family members varies among different plant species, with 39 identified in the *Arabidopsis* genome, 19 in rice [28], 48 in cotton [29], 61 in banana [30], and 33 in litchi [31] genomes, implying the diversity of *CASPL* family composition across species. Moreover, it has been found that *CASPL* family genes play an important role in abiotic stress, including salt tolerance [32,33,34] and cold tolerance [35,36]. However, the research on the function of plant *CASPL* genes is mainly focused on model plants such as *Arabidopsis thaliana* and rice, and there is no research reported on *PatCASPL* genes in *P. cablin*. Previous transcriptome data showed that *PatCASPLs* can respond to the allelochemical p-HBA, suggesting that the Casparian strip may play an important role in continuous cropping obstacles [16]. How many *PatCASPL* family genes are there in *P. cablin*? Which *CASPL* genes are involved in the continuous cropping obstacles of *P. cablin*? These questions need to be further answered.

In this study, based on the whole-genome data of *P. cablin*, a total of 156 *PatCASPL* family members were screened and identified through a bioinformatics analysis, and their protein physicochemical properties, chromosome distribution, promoter cis-acting elements, and evolutionary expression characteristics were analyzed in detail. Furthermore, 57 key *CASPL* candidate genes involved in the continuous cropping obstacles of *P. cablin* were screened and identified, via an analysis of the transcriptome data of *P. cablin* treated with p-HBA. The results of this study provide a theoretical basis for exploring the functions of the *PatCASPL* gene family and its role in continuous cropping obstacles.

## 2. Results

### 2.1. Identification of PatCASPL Gene Family Members and Analysis of Protein Physicochemical Properties in P. cablin

According to the 39 *CASPL* protein sequences of *A. thaliana*, 176 and 168 *CASPL* candidate genes were screened preliminarily in the *P. cablin* genome database by using the BLASTP [37] and HMM [38] alignment search methods, respectively. After further comparison and analysis using the SMART online program on the NCBI website (https://blast.ncbi.gov/ accessed on 10 May 2023), a total of 156 members of the *PatCASPL* gene family were finally identified. The physicochemical properties of the 156 *CASPL* protein sequences were analyzed, and the isoelectric point and molecular weight of the *PatCASPL* protein were predicted using the ExPASy [39] online analysis tool (Table 1). The results showed that the number of amino acids of *PatCASPL* protein varied from 102 (*PatCASPL5C8*) to 365 (*PatCASPL1A6*). The molecular weight of *PatCASPL* protein was between 11,043.99 and 39,579.28 Da, including 43 acidic proteins (pI ≤ 7) and 113 basic proteins (pI ≥ 7). The aliphatic index ranged from 64.93 to 127.1. The theoretical pI of the 156 *CASPL* proteins ranged from 4.93 to 10.14. In the *CASPL* gene family, the instability index of 40 *CASPL* genes is greater than 40, which highlights an unstable protein; the instability index of 116 *CASPL* genes was less than 40, which highlights stable proteins. The grand average of hydropathicity (GRAVY) of the *PatCASPL2B4*, *PatCASPL2B2*, *PatCASPL1F10*, *PatCASPL2B5*, *PatCASPL4D12*, *PatCASPL1A8*, *PatCASPL1F11*, *PatCASPL2B8*, *PatCASPL4D3*, *PatCASPL2B1*, *PatCASPL1F9*, *PatCASPL1A7*, *PatCASPL4D4*, *PatCASPL1A9*, *PatCASPL2B3*, *PatCASPL1B1*, and *PatCASPL3A3* proteins was less than zero, implying that they are hydrophilic proteins; the average hydrophilicity of the remaining 139 *CASPL* genes was greater than zero, indicating that they were hydrophobic proteins.

A secondary structure analysis of the *PatCASPL* protein in *P. cablin* using the SOPMA online tool [40] showed that the *PatCASPL* protein contained four types of structures: Alpha helix (α-helix, 22.09~71.31%), Beta turn (β-turn, 3.88~53.44%), random coil (1.06~10.4%), and extended strand (11.48~53.91%) (Table 2).

A subcellular localization prediction analysis showed that the *PatCASPL* protein was mainly distributed on the cell membrane, accounting for 75%, and the possibility of prediction analysis on the cell membrane was between 50.31% and 100%. Among them, *PatCASPL1C9*, *PatCASPL4D3*, *PatCASPL4D6*, *PatCASPL1C2*, *PatCASPL1C1*, *PatCASPL1C7*, *PatCASPL1C5*, and *PatCASPL4D4* gene distribution predicted the possibility of distribution on the cell membrane as high as 100%. In addition, 18 *PatCASPL* proteins were only distributed in the nucleus, with a probability of 51.39% to 98.88%. Eight gene proteins (*PatCASPL4D11*, *PatCASPL4D7*, *PatCASPL1A6*, *PatCASPL5B3*, *PatCASPL4D8*, *PatCASPL1B2*, *PatCASPL1A8*, *PatCASPL5B4*) were distributed on the chloroplast, and the probability ranged from 49.45% to 86.61%. *PatCASPL2B5*, *PatCASPL2B7*, and *PatCASPL2B8* were distributed on peroxisomes, with a probability of 49.45% to 95%. The different subcellular localization of *PatCASPL* family proteins indicates that the gene family may play different biological functions and mainly act on the cell membrane, which is consistent with the characteristics of membrane proteins. These prediction results provide a reference for subsequent experiments.

### 2.2. Genetic Characterization and Phylogenetic Analysis of PatCASPL Gene Family Members in P. cablin

**Figure 1:**
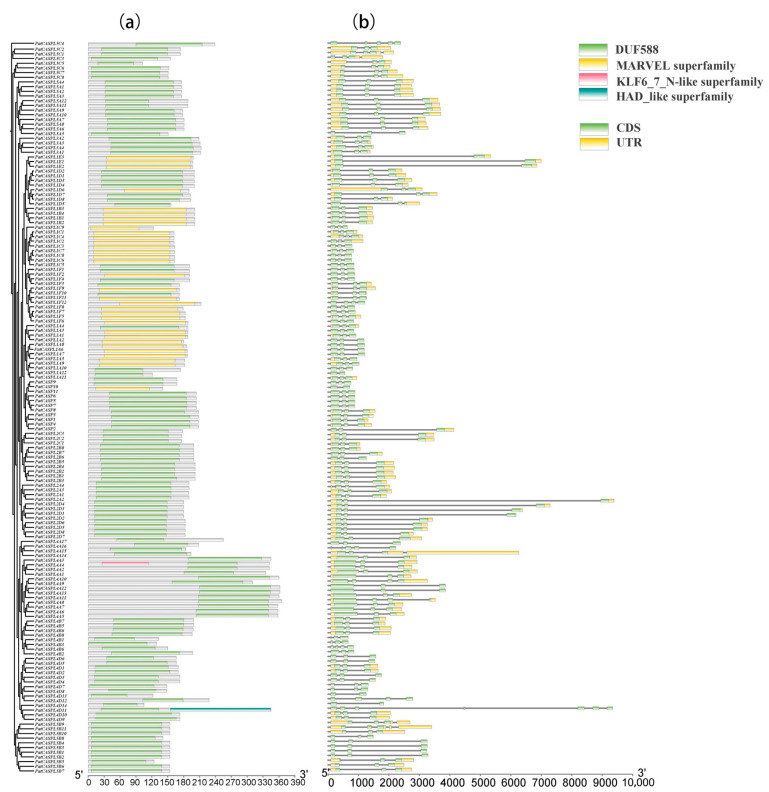
Conserved domain (**a**) and gene structure (**b**) of *CASPL* family members in *Pogostemon cablin*.

**Figure 2:**
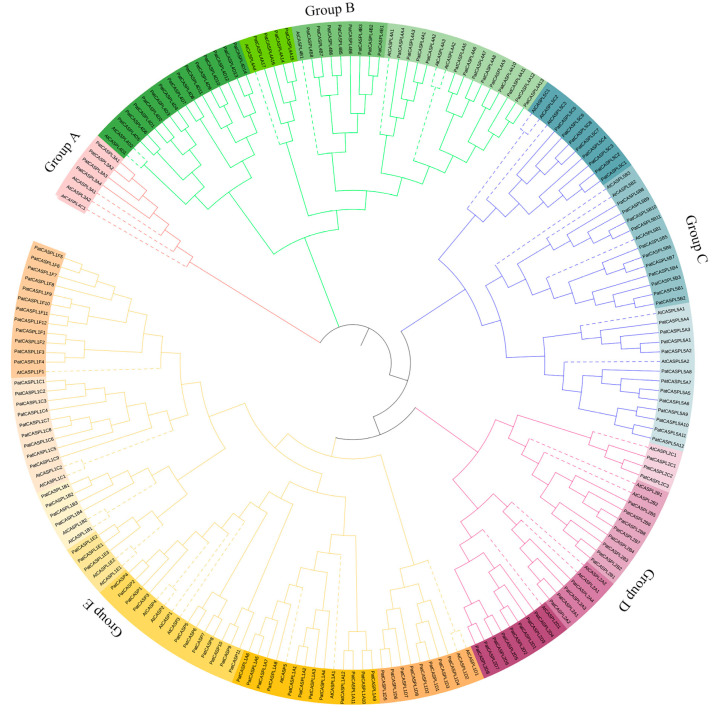
Phylogenetic trees of *CASPL* genes in *P. cablin* (Pat) and *Arabidopsis* (At). The phylogenetic tree of *CASPL* protein in *P. cablin* and *A. thaliana* was constructed with MEGA11 software using maximum likelihood method, and then visualized with the ITOL online tool. Different subfamilies are represented by branches and frames of different colors.

Based on the sequences of the *PatCASPL* gene family of *P. cablin*, the introns/exons and conserved motifs [41] were analyzed (Figure 1). A gene structure analysis showed that *PatCASPL* gene family members contained two to seven exons and two to eight introns. The members of the same subcluster have the same exons/introns, and this highly conserved gene structure affects the phylogenetic evolution relationship. Pfam (http://pfam.xfam.org/ accessed on 31 March 2023) and Batch CD-Search (https://www.ncbi.nlm.nih.gov/ accessed on 17 May 2023) were used to test whether the *PatCASPL* genes contained a complete domain, and the results showed that 156 *PatCASPL* gene family members contained DUF588 or MARVEL conserved domains. The two domains are selectively distributed in specific phylogenetic tree branches, showing the structural similarity between proteins in the same group. *PatCASP10*, *PatCASPL1A3*, *PatCASPL1C9*, *PatCASPL1F2*, and the other 34 genes containing the conserved domain of MARVEL were distributed in the E-subcluster (Group E) of the phylogenetic tree, and the remaining 122 genes containing the conserved domain of DUF588 were distributed in other subclusters of the phylogenetic tree (Figure 2). A subcellular localization prediction analysis showed that 25 genes, including *PatCASPL1C1*, *PatCASPL1E3*, *PatCASPL1F2*, and *PatCASPL1D6*, in 34 genes containing the MARVEL conserved domain were distributed on the cell membrane, and eight genes, including *PatCASPL1A5*, *PatCASPL1A8*, *PatCASPL1B4*, and *PatCASPL1B2,* were distributed on the chloroplast.

In addition to the DUF588 structure, *PatCASPL4A4* and *PatCASPL4D11* also contain the KLF6_7_N-like superfamily and HAD_like superfamily, respectively, suggesting that these two genes may have multiple functions. The analysis found that the function of the conserved domain of DUF588 has not been identified, containing a conserved arginine and aspartic acid, which constitutes a site that may have catalytic activity [42]. It has been reported that some scholars have extended phylogenetic analysis beyond the plant kingdom and found that there is conservation between the *CASPL* and MARVEL protein families, and conserved residues are located in transmembrane domains, indicating that these domains are involved in the localization of *CASPL* [21].

In order to explore the phylogenetic relationship of the *PatCASPL* gene family in *P. cablin*, the phylogenetic tree of 195 *CASPL* protein sequences in *P. cablin* and *A. thaliana* was constructed using the MEGA11 software [43]. The results showed that 195 *PatCASPL* and *AtCASPL* members could be divided into five subclusters, among which the A-subcluster (Group A) had the largest number of members, containing 15 *AtCASPL* members and 59 *PatCASPL* members. The B-Subcluster (Group B) contains six *AtCASPL* members and 23 *PatCASPL* members. The C-subcluster (Group C) contains seven *AtCASPL* members and 39 *PatCASPL* members. The number of members in the D-subcluster (Group D) is the lowest, containing only four *AtCASPL* members and three *PatCASPL* members. The E-subcluster (Group E) contains eight *AtCASPL* members and 31 *PatCASPL* members (Figure 2). The proportion of *AtCASPL* members to *PatCASPL* members in each cluster was 1:1~1:3.9, indicating that the *CASPL* genes in the same subcluster of *P. cablin* and *A. thaliana* may be derived from the same ancestor, and the chromosome doubling event of *P. cablin* led to the expansion of the number of *PatCASPL* family genes in *P. cablin*.

The E-subcluster (Group E) contains 11 genes with the highest homology with *AtCASP*. *AtCASP1-5* was identified as a gene related to the formation of *Arabidopsis* Casparian strip in *A. thaliana*. The function of *AtCASPL* protein in other subclusters has not been reported in depth. The genes distributed in the B-subcluster (Group B) may be involved in the response to abiotic stress. *At3g55390* (*AtCASPL4C1*) has been reported to be induced by low-temperature and negatively regulates plant growth [35]. Therefore, it is speculated that *PatCASPLs* with high homology may have similar functions. Up to now, the biological function of most *PatCASPL* genes in *P. cablin* is still unclear. However, more and more *CASPL* genes of *Arabidopsis* have been functionally characterized. Therefore, the clustering and comparison of *PatCASPL* proteins and *AtCASP* proteins can predict their functions through a homologous analysis.

### 2.3. Analysis of Cis-Acting Elements of PatCASPL Gene Family in P. cablin

**Figure 3:**
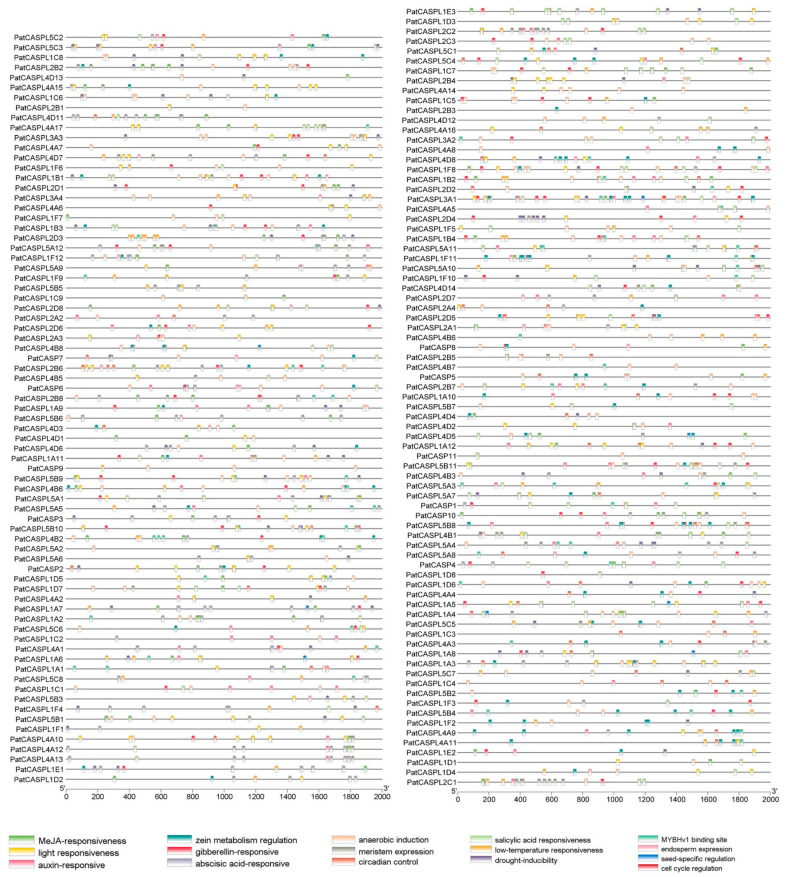
Cis-acting elements of *CASPL* family members in *P. cablin*. The 2000 bp promoter sequences of *P. cablin CASPL* genes contain a variety of cis-acting elements, including photo responsive elements, hormone responsive elements, drought, low-temperature, anaerobic, wound and other response elements, as well as specific elements of meristem, seed, and endosperm.

In order to explore the *PatCASPL*-involved signal regulation pathways, the cis-acting elements of the *PatCASPL* gene were analyzed [44]. A variety of different cis-elements were identified in the 2000 bp sequence upstream of the initiation codon of 156 *PatCASPL* gene families in *P. cablin*. Among them, the light response element is the most frequent in the *CASPL* gene, and 138 of the 156 genes contain light response elements, which is the most in the *PatCASPL* gene family, suggesting that the *PatCASPLs* may be involved in regulating the photomorphogenesis of *P. cablin* (Figure 3). Among the 156 members of the *PatCASPL* family, 152 members contained hormone-responsive elements, including 135 for abscisic acid, 88 for methyl jasmonate, 80 for gibberellin, 61 for auxin, and 61 for salicylic acid. There are 70 and 56 *PatCASPL* genes containing drought and low-temperature response elements, respectively, indicating that the *PatCASPLs* may play an important role in hormone regulation and the mitigation of stress. The cis-elements of different genes in the same subcluster of the *PatCASPL* gene family are not the same, suggesting that different *PatCASPLs* may play different functions in different growth, development, and stress response processes of *P. cablin*.

### 2.4. Chromosome Localization of PatCASPL Gene Family in P. cablin

**Figure 4:**
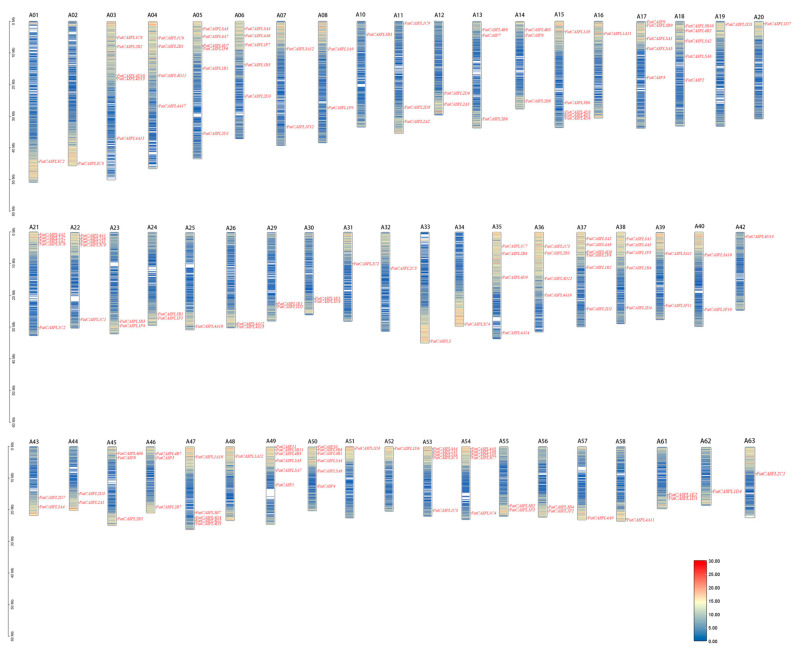
Chromosomal mapping of *CASPL* family genes in *P. cablin*. The leftmost scales represent the chromosome length; A01–A63 represent the names of 57 chromosomes of mustard. There is no *PatCASPL* gene on chromosomes 9, 27, 28, 41, 59, and 60. Blue indicates low gene density and red indicates high gene density.

A phylogenetic analysis showed that the *PatCASPL* gene family members were distributed in five subclusters, and their distribution showed an apparent chromosome preference, essentially distributed at both ends of the chromosome. Gene density information was obtained and analyzed using a gene density profile tool. The results of the gene density analysis (Figure 4) showed that the gene density of the 21 chromosome front segments of the *CASPL* gene was low, and the gene density of the back end was high. The gene density of the front and back ends of the remaining chromosomes was high, and the gene density of the middle was low. Twelve genes such as *PatCASPL4A15*, *PatCASPL4A1*, *PatCASPL2D1*, and *PatCASPL2D3* were distributed in the low-gene-density region, and 92% of the genes were located in the high-gene-density region.

A total of 156 *CASPL* genes identified in the whole genome of *P. cablin* were distributed on 57 of 63 chromosomes of *P. cablin* (Figure 4). The number of *CASPL* genes on each chromosome ranged from zero to six (Table 3). Among them, chromosomes 5, 37, 49, and 50 were the most distributed members, with six *CASPL* genes; five *CASPL* genes were distributed on chromosomes 6, 15, 17, 18, 21, 22, 38, 47, 53, and 54, respectively. Four *CASPL* genes were distributed on chromosomes 4, 35, and 36. There are one to three *CASPL* genes on another 40 chromosomes, while there is no *PatCASPL* gene on chromosomes 9, 27, 28, 41, 59, and 60. It is speculated that the number of *CASPL* members on each chromosome is not related to chromosome size. In addition, no tandem duplication was found in the *PatCASPL* gene family of *P. cablin*.

### 2.5. Expression Analysis of PatCASPL Gene Family in P. cablin

**Figure 5:**
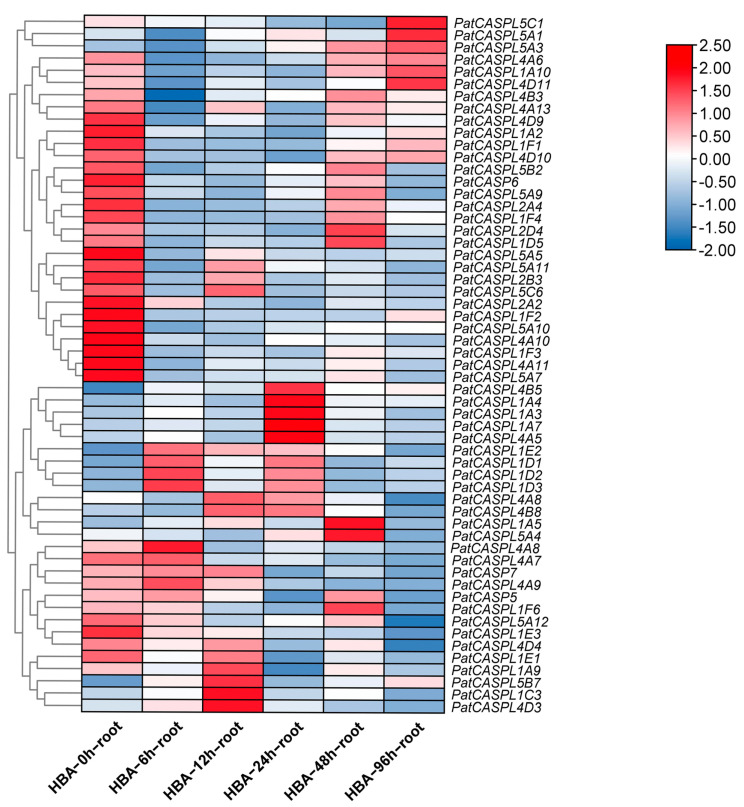
Expression of *PatCASPL* gene in *P. cablin* at different treatment stages of p-HBA.

In order to screen and identify the potential candidate genes of *P. cablin* in response to continuous cropping obstacles, the root transcriptome database (NCBI accession number PRJNA850618) of different periods (0 h, 6 h, 12 h, 24 h, 48 h, and 96 h) of p-HBA treatment was constructed in the previous study [16]. Cluster heat maps of 57 *CASPL* gene family transcripts were screened and constructed using the FPKM value of transcriptome data (Figure 5). The results showed that the *CASPL* genes in response to p-HBA could be divided into two types, namely p-HBA inhibited expression, and p-HBA promoted expression. In the first type, compared with the control (0 h), the expression levels of genes (*PatCASPL1A2*, *PatCASPL1F2*, *PatCASPL2A2*, *PatCASPL4A10*, *PatCASPL4A11*, *PatCASPL4D9*, *PatCASPL5A5*, *PatCASPL5A7*, *PatCASPL5A10*, and *PatCASP6*) were significantly down-regulated at 6 h, 12 h, and 24 h, while the expression levels of some genes (*PatCASPL1A10*, *PatCASPL1D5*, *PatCASPL2D4*, *PatCASPL5C1*, *PatCASPL5A1*, and *PatCASPL5A3*) were up-regulated at 48 h or 96 h, indicating that p-HBA inhibited the expression of these genes in the early stage, and the inhibitory effect gradually weakened over time or p-HBA consumption. In the second type, p-HBA treatment can promote the expression of *CASPL* genes such as *PatCASPL1A3*, *PatCASPL1A4*, *PatCASPL1A5*, *PatCASPL1C3*, *PatCASPL1D3*, *PatCASPL4A5*, *PatCASPL4B5*, *PatCASPL4A8*, and *PatCASPL5A4*, although the response time is different. Specifically, the gene expression levels of *PatCASPL1D3* and *PatCASPL4A8* peaked at 6 h and then decreased. The gene expression levels of *PatCASPL5B7*, *PatCASPL4D3*, and *PatCASPL1C3* were the highest at the 12 h treatment period. The gene expression levels of *PatCASPL4B5*, *PatCASPL1A4*, *PatCASPL1A3*, *PatCASPL1A7*, and *PatCASPL4A5* were the most significantly up-regulated at 24 h, while the gene expression levels of *PatCASPL2D4*, *PatCASPL5A4,* and *PatCASPL1A5* were at the maximum at 48 h. The gene expression of *PatCASPL4D9*, *PatCASPL1A2*, *PatCASPL1F1*, *PatCASP6*, *PatCASPL2A4*, *PatCASPL5A5*, *PatCASPL2B3*, *PatCASPL2A3*, *PatCASPL5A10*, *PatCASPL5A7*, *PatCASPL4A10*, *PatCASPL4A11*, *PatCASPL1F2*, *PatCASPL1F3*, and *PatCASPL1E3* showed a downward trend. The gene expression of *PatCASPL4B3* was most significantly down-regulated at 6 h, while the gene expression levels of *PatCASPL4D4* and *PatCASPL5A12* were significantly down-regulated at 96 h. The above 57 genes were highly expressed in their corresponding periods.

In the first type, the promoter regions of the significantly down-regulated genes at 6 h, 12 h, and 24 h contained abscisic acid-responsive elements, suggesting that their response to p-HBA may involve ABA signaling. Except *PatCASPL5A3,* which is distributed on chloroplasts, other genes are distributed on cell membranes, *PatCASPL1A2* and *PatCASPL1A10* contain MARVEL conserved domains, and other genes contain DUF588 conserved domains. In the second type, the promoter regions of *PatCASPL1A9*, *PatCASPL1E1*, *PatCASPL5A12*, *PatCASPL4D4*, and *PatCASPL4A8*, which were down-regulated in expression, contained drought response elements; *PatCASPL4A8* was distributed in the nucleus, and the other genes were distributed on the cell membrane. In addition to *PatCASPL1E1* containing the MARVEL conserved structure, other genes contained the DUF588 conserved domain. In summary, 34 genes containing the MARVEL conserved domain were more likely to be distributed on the cell membrane and contained a large number of MYB binding sites. It is speculated that they may indirectly affect the structure of the Casparian strip. In addition, the *PatCASPL* gene family of *Pogostemon cablin* also contains many other types of cis-acting elements, among which the number of light-responsive elements is the largest. It can be judged that the *CASPL* family genes may have biological functions on the regulation of circadian rhythm and photomorphogenesis.

The expression of *PatCASPLs* changed after p-HBA treatment, suggesting that it may play an important role in the response of *P. cablin* to p-HBA treatment. It can be used as a candidate in gene screening for further resistance research and functional analysis. It is speculated that p-HBA may affect the expression of these genes, thereby affecting the formation and integrity of the Casparian strip, reducing the tolerance of *P. cablin* to stress, and ultimately leading to continuous cropping obstacles. The specific functions of these genes need to be further verified.

## 3. Discussion

In this study, 156 *CASPL* genes were identified via a bioinformatics analysis based on the genome data of *P. cablin*. The number of *PatCASPLs* is much larger than those of *A. thaliana* (39) [21], rice (19) [28], cotton (48) [29], banana (61) [30], and litchi (33) [31]. The number of gene family members among species is affected by the number of genome doublings, tandem repeats, and natural selection [45]. Long-terminal repeat retrotransposons (LTR-RTs) can proliferate rapidly in the host, thus affecting the expression of genes. It has been reported that the transposition and amplification of LTR-RTs is an important factor in the expansion of the plant genome [46,47]. Previous studies have found that the genome of *P. cablin* had a chromosome doubling event (3.3 million years ago) and an LTR-RT insertion event (1.1 million years ago) [48]. This also explains why the number of *PatCASPL* family members in *P. cablin* is far more than that of other species and is consistent with the results that the number of *AtCASPL* members and *PatCASPL* members in each cluster is 1:1~1:3.9. The conserved domains of the *CASPL* gene family are also various in different species. The MARVEL domain is present in the *CASPL* gene family of *A. thaliana*, banana, and litchi [21]; however, it has not been reported in rice, indicating that the number and domain of *CASPL* gene family members in different species are also diverse.

In this research, the cis-elements analysis showed that the *PatCASPL* gene family has more hormone response and stress response elements, and it is speculated that the gene family may play an important role in regulating growth and development and stress tolerance. Previous studies have found that the expression of *TaCASPLs* can be induced in response to salt stress, osmotic stress, and calcium ion stress, and their expression is inhibited under low-temperature stress [49]. Moreover, Liu et al. [32] found that the *SbCASP-LP1C1* gene was involved in the formation of an extracellular barrier and improved the salt tolerance of sweet sorghum. Similarly, Rushil et al. [50] showed that the *OsCASPL1* protein has a certain role in salt stress tolerance. Yang et al. [35] identified a cold-inducible protein *ClCASPL* gene in watermelon. In addition, *AtCASPL4C1*, the homologous gene in *A. thaliana*, plays an important role in cold tolerance [51]. The above results suggest that the *PatCASPL* gene may also play a key role in abiotic stress responses.

In agricultural practice, the continuous planting of *P. cablin* on the same land will lead to changes in the soil’s physical and chemical properties [52,53], the microbial community structure, the aggravation of soil diseases, and more serious allelopathic autotoxicity, resulting in serious continuous cropping obstacles [54,55,56,57]. Previous studies have found that p-HBA is the main allelochemical that induces continuous cropping obstacles of *P. cablin* [16,58]. In this study, 30 down-regulated candidate genes and 27 up-regulated candidate genes were identified by analyzing the transcriptome data of *P. cablin* roots treated with p-HBA. The down-regulated expression of candidate genes may contribute to the incomplete Casparian strip, and then to the diffusion of the allelochemicals through the incomplete Casparian strip and long-distance transportation in a vascular bundle. These allelochemicals cause plant metabolic disorder, affecting plant growth and development, finally leading to the occurrence of *P. cablin* continuous cropping obstacles. The up-regulated expression of some genes may partially compensate for the incomplete Casparian strip and slow down the entry of allelochemicals into plant vascular bundles. The functions of these candidate genes need to be further verified. 

## 4. Materials and Methods

### 4.1. Genome-Wide Identification of CASPL from P. cablin

The *Arabidopsis* genome data were downloaded from the TAIR website (https://www.arabidopsis.org/ accessed on 24 March 2023.), and the patchouli genome sequence file, protein sequence file, and gene structure annotation file were downloaded from the GSA website (https://ngdc.cncb.ac.cn/gsa/ accessed on 24 April 2023). The amino acid sequences of 39 *CASPL* proteins in *A. thaliana* were extracted using the TBTools (v1.120) software [59]. To obtain homologous sequences, the *Arabidopsis CASPL* gene protein sequences were aligned with the patchouli protein data (*e*-value < 1× 10^−5^) using BLASTP [60], followed by dereplication. At the same time, the Pfam (number PF04535) of *CASPL* was obtained in the InterPro [61] database (www.ebi.ac.uk/interpro accessed on 24 March 2023), and its hidden Markov model (HMM) file was downloaded. The HMM [62] file of *CASPL* was used as input and compared in the TBtools software to obtain candidate gene IDs and extract candidate gene protein sequences. The amino acid sequences of the candidate proteins obtained via the two alignment methods were merged and the repeat sequences were deleted. The conserved domain of the *CASPL* gene was further analyzed, the candidate genes with an incomplete or missing *CASPL* domain were deleted, and the *PatCASPL* genes were finally obtained. The Expasy [63] website (https://www.expasy.org// accessed on 19 July 2023) was used to predict the number of amino acids and the protein molecular weight of *CASPL* family members. The subcellular localization of *CASPL* family proteins was predicted using the Plant-mPLoc [64,65] online software (http://www.csbio.sjtu.edu.cn/bioinf/Cell-PLoc2/ accessed on 11 September 2023) and the YLoc [66] online software (https://abi-services.cs.uni-tuebingen.de/yloc/webloc.cgi accessed on 12 November 2023), and the secondary structure of the *CASPL* family proteins was predicted and analyzed using the SOPMA [40] online software (https://npsa-prabi.ibcp.fr// accessed on 11 September 2023).

### 4.2. Genetic Characteristics and Phylogenetic Analysis

The position information of the exons, introns, and UTR of *CASPL* family genes was obtained from the patchouli genome annotation information (gff) file, and the *CASPL* gene structure map was drawn using the TBtools software. We used MEME [67] online (http://meme-suite.org/ accessed on 17 May 2023) to analyze the conserved motifs of *CASPL* proteins. The maximum number and the length of motifs was set at eight and 6–50 amino acids, respectively. The TBtools software was used for a visual analysis. Using the original sequence, the PF04535.15 domain was found in the Pfam [68] database (http://pfam.xfam.org/ accessed on 31 March 2023). The domain information was extracted and the *CASPL* protein sequence from *P. cablin* and *A. thaliana* were compared using the muscle program [69]. The results were compared with the ProtTest to predict the optimal model [70]. The phylogenetic tree of the *CASPL* protein in *P. cablin* and *A. thaliana* was constructed with the MEGA11 software using the maximum likelihood method, and then visualized with the ITOL online tool (itol.embl.de/). The promoter sequences of the *CASPL* gene family members in *P. cablin* were extracted with TBtools, and the promoter cis-elements were predicted and analyzed with the PlantCARE [71] online program (http://bioinformatics.psb.ugent.be/webtools/plantcare/html/ accessed on 8 August 2023).

### 4.3. Chromosomal Localization Analysis

Based on the patchouli genome annotation file, the length information of 63 chromosomes and the location information of all *PatCASPL* genes on chromosomes were obtained, followed by visualization. The gene density profile tool of the Tbtools software was used to obtain the gene density information of the *PatCASPL* gene. And the location information and distance relationship of all patchouli *PatCASPL* gene family members were marked on chromosomes via the TBtools software.

### 4.4. Expression Analysis

According to the previous transcriptome study [6,72] of *P. cablin* roots, the transcriptome data of *P. cablin* roots at different time points (0 h, 6 h, 12 h, 24 h, 48 h, and 96 h) under p-hydroxybenzoic acid (p-HBA) treatment were obtained. The above raw data have been uploaded to the NCBI (https://www.ncbi.nlm.nih.gov/ accessed on 28 July 2023) sequence read file (SRA) (accession number PRJNA850618) [11]. The transcript expression of *CASPL* genes were extracted from the above transcriptome data, and were preliminarily screened using the Excel software of Microsoft office 2019. After that, the selected data were further processed with the TBtools (v1.120) software and the clustering heat map was drawn.

## 5. Conclusions

In summary, in order to explore the biological function of the *PatCASPL* gene family and its role in continuous cropping obstacles, the members of the *PatCASPL* family were identified and analyzed at the genome-wide level. The composition, physicochemical properties, evolutionary relationship, and potential biological function of *PatCASPL* family members were characterized and figured out. These results provide an important theoretical basis for further exploring the biological function of the *PatCASPL* gene family and its role in continuous cropping obstacles.

## Figures and Tables

**Table 1:** Physicochemical properties of *CASPL* gene family proteins in *Pogostemon cablin*.

Gene ID	Gene Name	Amino Acids	Molecular Weight (DA)	Theoretical pI	Instability Index	Aliphatic Index	Gravy
*Pat_A01G155900*	*PatCASPL5C2*	174	18,737.06	6.08	40.99	119.37	0.87
*Pat_A02G232800*	*PatCASPL5C3*	155	16,722.93	8.43	38.68	127.10	1.12
*Pat_A03G065000*	*PatCASPL1C8*	163	17,595.99	8.88	35.43	113.80	0.84
*Pat_A03G097400*	*PatCASPL2B2*	202	21,785.90	9.68	23.44	111.44	0.66
*Pat_A03G197700*	*PatCASPL4D10*	173	19,008.18	9.12	31.33	110.98	0.44
*Pat_A03G202900*	*PatCASPL4D13*	122	13,830.31	9.10	26.09	104.84	0.62
*Pat_A03G283300*	*PatCASPL4A15*	184	20,313.68	9.44	45.11	108.64	0.49
*Pat_A04G068200*	*PatCASPL1C6*	163	17,533.92	8.88	32.79	115.58	0.84
*Pat_A04G099600*	*PatCASPL2B1*	202	21,785.90	9.68	23.44	111.44	0.66
*Pat_A04G201600*	*PatCASPL4D11*	345	39,281.21	8.73	33.56	84.43	−0.14
*Pat_A04G257700*	*PatCASPL4A17*	255	28,561.81	9.51	56.87	92.55	−0.11
*Pat_A05G029500*	*PatCASPL3A3*	211	22,711.54	9.47	35.96	109.05	0.46
*Pat_A05G058400*	*PatCASPL4A7*	358	38,903.50	8.55	57.00	65.39	−0.42
*Pat_A05G090400*	*PatCASPL4D7*	148	16,269.20	8.79	35.37	115.41	0.66
*Pat_A05G091800*	*PatCASPL1F6*	183	20,103.52	9.32	29.58	100.87	0.65
*Pat_A05G144200*	*PatCASPL1B1*	201	21,547.42	9.47	39.12	113.98	0.56
*Pat_A05G199200*	*PatCASPL2D1*	180	20,062.37	8.09	30.02	92.17	0.53
*Pat_A06G029300*	*PatCASPL3A4*	213	23,033.91	9.62	41.45	108.03	0.41
*Pat_A06G056900*	*PatCASPL4A6*	358	39,077.65	7.73	55.41	65.11	−0.42
*Pat_A06G090000*	*PatCASPL1F7*	179	19,708.02	9.32	30.94	99.27	0.65
*Pat_A06G141700*	*PatCASPL1B3*	201	21,663.45	9.12	39.24	112.54	0.51
*Pat_A06G179600*	*PatCASPL2D3*	180	20,071.38	8.10	27.79	92.17	0.53
*Pat_A07G098800*	*PatCASPL5A12*	188	20,282.53	8.45	63.79	108.94	0.34
*Pat_A07G167500*	*PatCASPL1F12*	172	18,802.58	9.63	28.60	114.07	0.88
*Pat_A08G099100*	*PatCASPL5A9*	178	18,825.80	6.05	45.93	103.71	0.60
*Pat_A08G164800*	*PatCASPL1F9*	172	18,754.51	9.72	27.48	119.19	0.94
*Pat_A10G028900*	*PatCASPL5B5*	124	13,552.02	6.50	23.84	114.92	1.05
*Pat_A11G002900*	*PatCASPL1C9*	123	13,753.60	9.47	33.02	123.66	0.90
*Pat_A11G093600*	*PatCASPL2D8*	183	20,488.08	6.53	30.97	104.97	0.63
*Pat_A11G130300*	*PatCASPL2A2*	190	20,283.41	5.34	33.29	97.68	0.48
*Pat_A12G091200*	*PatCASPL2D6*	183	20,446.97	5.27	31.60	106.56	0.65
*Pat_A12G123600*	*PatCASPL2A3*	190	20,297.44	5.34	33.54	98.16	0.48
*Pat_A13G026600*	*PatCASPL4B8*	196	21,649.67	5.74	43.43	94.18	0.004
*Pat_A13G039300*	*PatCASP7*	204	21,541.21	6.81	22.06	115.78	0.74
*Pat_A13G129500*	*PatCASPL2B6*	199	21,402.50	9.56	26.51	112.71	0.64
*Pat_A14G027100*	*PatCASPL4B5*	199	21,921.03	5.74	43.12	94.72	−0.005
*Pat_A14G039900*	*PatCASP6*	204	21,622.37	7.69	21.23	117.7	0.74
*Pat_A14G121300*	*PatCASPL2B8*	199	21,423.56	9.66	25.27	114.67	0.63
*Pat_A15G045000*	*PatCASPL1A9*	182	19,520.78	8.88	19.13	112.64	0.61
*Pat_A15G155900*	*PatCASPL5B6*	154	16,555.60	8.34	26.73	112.21	1.02
*Pat_A15G171700*	*PatCASPL4D3*	173	18,398.69	9.64	25.70	123.53	0.89
*Pat_A15G171900*	*PatCASPL4D1*	170	17,879.91	9.47	38.89	118.24	0.80
*Pat_A15G172200*	*PatCASPL4D6*	166	17,610.64	7.66	18.61	115.72	0.87
*Pat_A16G047300*	*PatCASPL1A11*	121	13,427.72	9.06	24.30	106.45	0.46
*Pat_A17G000600*	*PatCASP9*	167	18,014.18	5.46	30.63	121.02	0.95
*Pat_A17G012200*	*PatCASPL5B9*	154	16,555.67	8.60	33.09	118.44	1.005
*Pat_A17G030600*	*PatCASPL4B6*	150	16,487.81	5.30	41.08	86.67	0.02
*Pat_A17G063900*	*PatCASPL5A1*	176	18,963.21	9.20	44.76	92.56	0.65
*Pat_A17G094300*	*PatCASPL5A5*	151	15,959.66	4.99	35.49	115.03	0.92
*Pat_A17G121700*	*PatCASP3*	208	22,068.68	9.15	26.79	108.94	0.69
*Pat_A18G014400*	*PatCASPL5B10*	154	16,555.67	8.60	33.09	118.44	1.005
*Pat_A18G033100*	*PatCASPL4B2*	197	21,716.96	5.85	35.23	95.18	0.15
*Pat_A18G065500*	*PatCASPL5A2*	176	18,963.21	9.20	44.76	92.56	0.65
*Pat_A18G103000*	*PatCASPL5A6*	181	19,094.24	5.51	37.05	107.29	0.68
*Pat_A18G121000*	*PatCASP2*	208	22,014.65	9.37	27.61	106.63	0.68
*Pat_A19G009800*	*PatCASPL1D5*	156	16,721.60	6.82	47.56	114.42	0.56
*Pat_A20G008500*	*PatCASPL1D7*	193	20,509.26	7.74	31.53	122.80	0.81
*Pat_A21G011400*	*PatCASPL4A2*	342	37,157.91	7.22	57.64	76.90	−0.38
*Pat_A21G024900*	*PatCASPL1A7*	187	20,028.60	9.95	27.68	117.91	0.78
*Pat_A21G025000*	*PatCASPL1A2*	188	20,162.56	6.90	32.00	110.16	0.63
*Pat_A21G042100*	*PatCASPL5C6*	152	16,340.38	4.93	35.02	125.07	1.11
*Pat_A21G143800*	*PatCASPL1C2*	162	17,433.89	9.16	31.88	122.84	0.89
*Pat_A22G011800*	*PatCASPL4A1*	335	36,352.02	7.19	55.74	78.51	−0.33
*Pat_A22G025300*	*PatCASPL1A6*	186	19,849.45	10.14	25.57	122.20	0.86
*Pat_A22G025400*	*PatCASPL1A1*	190	20,421.00	6.90	33.71	111.58	0.68
*Pat_A22G042500*	*PatCASPL5C8*	151	16,212.31	4.93	31.17	126.56	1.18
*Pat_A22G142300*	*PatCASPL1C1*	162	17,433.89	9.16	30.17	122.22	0.89
*Pat_A23G122600*	*PatCASPL5B3*	154	16,693.77	7.60	33.21	109.03	0.93
*Pat_A23G137500*	*PatCASPL1F4*	191	20,833.48	8.87	29.87	114.40	0.58
*Pat_A24G110200*	*PatCASPL5B1*	154	16,661.71	7.60	29.81	110.91	0.94
*Pat_A24G124700*	*PatCASPL1F1*	191	20,851.55	8.77	30.75	114.40	0.59
*Pat_A25G140600*	*PatCASPL4A10*	360	38,979.39	5.79	58.93	71.86	−0.29
*Pat_A26G134800*	*PatCASPL4A12*	360	38,827.08	5.42	56.84	70.50	−0.30
*Pat_A26G135400*	*PatCASPL4A13*	362	38,985.23	5.42	57.11	70.39	−0.30
*Pat_A29G083500*	*PatCASPL1E1*	197	20,742.55	9.28	32.97	114.31	0.68
*Pat_A29G084000*	*PatCASPL1D2*	200	21,362.40	9.59	32.87	119.40	0.74
*Pat_A30G087000*	*PatCASPL1E3*	198	20,843.65	9.28	31.88	114.70	0.68
*Pat_A30G087100*	*PatCASPL1D3*	200	21,315.34	9.48	36.63	118.90	0.74
*Pat_A31G096200*	*PatCASPL2C2*	176	19,411.14	9.15	29.96	123.12	0.74
*Pat_A32G099900*	*PatCASPL2C3*	178	19,842.76	9.44	29.99	125.56	0.71
*Pat_B01G208300*	*PatCASPL5C1*	174	18,737.06	6.08	40.99	119.37	0.87
*Pat_B02G201600*	*PatCASPL5C4*	239	26,174.03	9.33	40.75	112.26	0.79
*Pat_B03G060000*	*PatCASPL1C7*	163	17,624.04	8.88	34.91	114.97	0.86
*Pat_B03G092600*	*PatCASPL2B4*	202	21,845.95	9.65	25.24	111.93	0.64
*Pat_B03G186600*	*PatCASPL4D9*	173	19,036.24	9.12	30.84	112.66	0.46
*Pat_B03G261500*	*PatCASPL4A14*	194	21,175.57	9.03	43.93	108.04	0.48
*Pat_B04G064500*	*PatCASPL1C5*	163	17,604.05	8.88	29.63	117.36	0.86
*Pat_B04G095000*	*PatCASPL2B3*	202	21,842.00	9.68	23.44	112.87	0.67
*Pat_B04G197000*	*PatCASPL4D12*	228	25,002.18	9.85	44.57	91.27	0.19
*Pat_B04G241200*	*PatCASPL4A16*	208	22,798.12	9.12	57.53	90.53	0.15
*Pat_B05G029100*	*PatCASPL3A2*	209	22,610.39	9.47	37.00	107.75	0.44
*Pat_B05G054900*	*PatCASPL4A8*	365	39,579.28	8.98	56.32	64.93	−0.41
*Pat_B05G085300*	*PatCASPL4D8*	148	16,255.17	8.79	37.18	115.41	0.66
*Pat_B05G086500*	*PatCASPL1F8*	213	23,110.08	9.57	28.73	93.52	0.55
*Pat_B05G131000*	*PatCASPL1B2*	201	21,571.40	9.47	40.29	112.04	0.53
*Pat_B05G177300*	*PatCASPL2D2*	180	20,062.37	8.09	30.02	92.17	0.53
*Pat_B06G029300*	*PatCASPL3A1*	212	22,919.85	9.84	39.47	108.54	0.44
*Pat_B06G055200*	*PatCASPL4A5*	358	38,998.60	8.25	56.75	65.67	−0.42
*Pat_B06G087000*	*PatCASPL1F5*	183	20,123.51	9.32	30.64	98.74	0.65
*Pat_B06G133700*	*PatCASPL1B4*	201	21,635.44	9.33	39.00	112.54	0.53
*Pat_B06G179700*	*PatCASPL2D4*	180	20,161.50	8.09	27.37	92.17	0.53
*Pat_B07G090500*	*PatCASPL5A11*	188	20,285.57	8.84	66.99	109.47	0.34
*Pat_B07G153300*	*PatCASPL1F11*	172	18,722.48	9.75	31.64	118.55	0.93
*Pat_B08G094100*	*PatCASPL5A10*	178	18,853.85	6.05	46.41	104.78	0.62
*Pat_B08G157700*	*PatCASPL1F10*	172	18,740.48	9.72	27.48	118.60	0.94
*Pat_B10G009400*	*PatCASPL4D14*	105	12,171.19	9.33	29.48	97.33	0.27
*Pat_B11G071200*	*PatCASPL2D7*	183	20,442.99	5.74	32.02	104.97	0.61
*Pat_B11G102300*	*PatCASPL2A4*	190	20,328.51	5.06	34.55	99.21	0.52
*Pat_B12G066000*	*PatCASPL2D5*	183	20,487.08	5.27	29.96	109.23	0.67
*Pat_B12G095400*	*PatCASPL2A1*	189	20,260.42	5.78	35.12	97.67	0.48
*Pat_B13G025200*	*PatCASPL4B6*	198	21,762.83	6.74	44.34	93.23	−0.002
*Pat_B13G036500*	*PatCASP8*	204	21,580.29	7.69	22.06	116.27	0.73
*Pat_B13G115900*	*PatCASPL2B5*	199	21,458.52	9.66	26.51	110.75	0.58
*Pat_B14G025500*	*PatCASPL4B7*	199	22,009.18	5.74	41.72	95.68	0.02
*Pat_B14G037300*	*PatCASP5*	204	21,606.37	7.69	21.23	118.19	0.76
*Pat_B14G109400*	*PatCASPL2B7*	199	21,368.48	9.56	26.51	114.67	0.65
*Pat_B15G046400*	*PatCASPL1A10*	182	19,477.75	8.50	18.54	114.78	0.66
*Pat_B15G144800*	*PatCASPL5B7*	154	16,569.62	8.34	26.73	112.86	1.01
*Pat_B15G158100*	*PatCASPL4D4*	173	18,382.65	9.64	28.24	121.27	0.86
*Pat_B15G158200*	*PatCASPL4D2*	170	17,868.88	9.26	41.73	117.65	0.81
*Pat_B15G158500*	*PatCASPL4D5*	166	17,651.73	8.47	18.61	116.33	0.85
*Pat_B16G041300*	*PatCASPL1A12*	174	19,290.61	9.28	34.81	99.25	0.41
*Pat_B17G001300*	*PatCASP11*	141	15,161.67	5.99	35.21	111.56	0.75
*Pat_B17G012900*	*PatCASPL5B11*	154	16,555.67	8.60	33.09	118.44	1.005
*Pat_B17G030600*	*PatCASPL4B3*	129	14,404.54	5.70	27.96	88.68	0.12
*Pat_B17G058000*	*PatCASPL5A3*	176	19,005.29	9.20	45.24	94.20	0.68
*Pat_B17G090900*	*PatCASPL5A7*	181	19,035.16	5.51	40.28	108.95	0.69
*Pat_B17G110800*	*PatCASP1*	208	22,040.67	9.37	27.80	108.03	0.68
*Pat_B18G001200*	*PatCASP10*	167	18,014.18	5.46	30.63	121.02	0.95
*Pat_B18G012300*	*PatCASPL5B8*	141	15,078.88	8.61	30.17	114.82	0.98
*Pat_B18G028600*	*PatCASPL4B1*	133	15,018.22	7.69	29.85	80.08	0.14
*Pat_B18G055600*	*PatCASPL5A4*	176	18,975.20	9.41	45.02	93.12	0.63
*Pat_B18G089000*	*PatCASPL5A8*	181	19,025.13	5.10	36.78	107.85	0.70
*Pat_B18G103200*	*PatCASP4*	208	22,111.72	9.15	27.61	105.67	0.65
*Pat_B19G008300*	*PatCASPL1D8*	193	20,528.17	6.26	33.63	119.74	0.79
*Pat_B20G008600*	*PatCASPL1D6*	190	20,477.88	8.84	29.53	98.11	0.37
*Pat_B21G010200*	*PatCASPL4A4*	342	36,900.68	8.14	54.87	77.75	−0.33
*Pat_B21G023100*	*PatCASPL1A5*	187	20,041.69	9.95	23.85	120.00	0.82
*Pat_B21G023200*	*PatCASPL1A4*	188	20,159.55	6.41	31.90	111.70	0.62
*Pat_B21G039100*	*PatCASPL5C5*	102	11,043.99	7.68	41.53	112.75	0.86
*Pat_B21G131300*	*PatCASPL1C3*	162	17,432.91	9.33	31.12	122.84	0.89
*Pat_B22G012300*	*PatCASPL4A3*	345	37,637.43	6.84	57.25	76.06	−0.34
*Pat_B22G025600*	*PatCASPL1A8*	180	19,425.94	9.93	26.11	121.33	0.80
*Pat_B22G025700*	*PatCASPL1A3*	188	20,273.76	6.90	34.33	111.70	0.63
*Pat_B22G041800*	*PatCASPL5C7*	151	16,228.31	4.93	34.08	125.89	1.17
*Pat_B22G135200*	*PatCASPL1C4*	162	17,417.89	9.16	31.88	123.46	0.91
*Pat_B23G102000*	*PatCASPL5B2*	154	16,661.71	7.60	29.81	110.91	0.94
*Pat_B23G117100*	*PatCASPL1F3*	191	20,808.52	8.77	28.47	114.92	0.62
*Pat_B24G101400*	*PatCASPL5B4*	154	16,675.74	7.60	31.55	111.56	0.94
*Pat_B24G116200*	*PatCASPL1F2*	191	20,857.51	8.77	31.68	111.83	0.59
*Pat_B25G125300*	*PatCASPL4A9*	310	33,975.81	5.53	53.22	71.48	−0.23
*Pat_B26G124600*	*PatCASPL4A11*	360	39,014.24	5.59	58.05	68.89	−0.34
*Pat_B29G065200*	*PatCASPL1E2*	197	20,772.57	9.28	32.97	114.31	0.66
*Pat_B29G065600*	*PatCASPL1D1*	200	21,303.33	9.48	34.80	119.40	0.76
*Pat_B30G065400*	*PatCASPL1D4*	200	21,287.24	9.25	35.15	118.90	0.75
*Pat_B31G082800*	*PatCASPL2C1*	176	19,524.25	9.15	31.35	123.12	0.70

**Table 2:** Subcellular localization and protein secondary structure analysis of *CASPL* gene family proteins in *P. cablin*.

Gene Name	Subcellular Location	Prediction Probability %	Alpha Helix %	Extended Strand %	Beta Turn %	Random Coil %
*PatCASPL5C2*	Cell membrane	84.60	53.45	16.09	3.45	27.01
*PatCASPL5C3*	Cell membrane	77.10	55.48	14.84	2.58	27.10
*PatCASPL1C8*	Cell membrane	93.50	57.67	16.56	4.29	21.47
*PatCASPL2B2*	Cell membrane/Chloroplast/Peroxisome	51.80/39.00/5.00	61.39	15.35	3.96	19.31
*PatCASPL4D10*	Cell membrane	86.00	53.18	16.18	10.40	20.23
*PatCASPL4D13*	Cell membrane	83.00	69.67	13.93	4.92	11.48
*PatCASPL4A15*	Cell membrane	53.30	54.89	15.22	1.09	28.80
*PatCASPL1C6*	Cell membrane	93.40	57.06	18.40	4.29	20.25
*PatCASPL2B1*	Cell membrane/Chloroplast/Peroxisome	51.81/30.10/17.00	61.39	15.35	3.96	19.31
*PatCASPL4D11*	Chloroplast	90.10	38.26	18.84	6.96	35.94
*PatCASPL4A17*	Nucleus	96.50	45.88	12.55	6.67	34.90
*PatCASPL3A3*	Nucleus	81.10	46.45	9.00	5.21	39.34
*PatCASPL4A7*	Nucleus	91.00	30.17	13.69	6.42	49.72
*PatCASPL4D7*	Chloroplast	86.10	54.05	20.27	6.08	19.59
*PatCASPL1F6*	Cell membrane	92.40	53.55	17.49	2.19	26.78
*PatCASPL1B1*	Chloroplast/Nucleus	99.10/33.03	46.77	16.92	8.96	27.36
*PatCASPL2D1*	Cell membrane	75.00	56.67	17.78	2.78	22.78
*PatCASPL3A4*	Nucleus	81.08	47.42	8.92	3.76	39.91
*PatCASPL4A6*	Nucleus	92.60	27.93	14.80	3.63	53.63
*PatCASPL1F7*	Cell membrane	95.70	51.40	16.76	3.91	27.93
*PatCASPL1B3*	Chloroplast/Nucleus	98.90/32.93	51.74	12.94	6.97	28.36
*PatCASPL2D3*	Cell membrane	88.30	57.78	15.56	1.67	25.00
*PatCASPL5A12*	Cell membrane	85.90	37.77	15.96	7.98	38.30
*PatCASPL1F12*	Cell membrane	78.60	56.98	16.28	4.65	22.09
*PatCASPL5A9*	Cell membrane/Golgi apparatus/Nucleus	62.30/95.00/17	51.12	10.67	3.93	34.27
*PatCASPL1F9*	Cell membrane	55.20	57.56	15.7	4.65	22.09
*PatCASPL5B5*	Cell membrane	99.97	62.90	10.48	5.65	20.97
*PatCASPL1C9*	Cell membrane	100.00	41.46	30.89	4.07	23.58
*PatCASPL2D8*	Cell membrane	98.50	56.28	20.22	2.73	20.77
*PatCASPL2A2*	Cell membrane/Nucleus	92.65/28.00	55.79	17.89	1.58	24.74
*PatCASPL2D6*	Cell membrane/Chloroplast	98.50/28.00	57.38	18.03	3.83	20.77
*PatCASPL2A3*	Cell membrane/Nucleus	92.65/36.00	56.32	17.89	2.63	23.16
*PatCASPL4B8*	Cell membrane/Chloroplast/Cytoplasm/Mitochondrion/Nucleus	78.42/49.00/7.00/95.00/7.00	60.20	4.59	2.04	33.16
*PatCASP7*	Cell membrane/Golgi apparatus/Nucleus	80.48/16.00/23.00	50.49	17.65	3.92	27.94
*PatCASPL2B6*	Mitochondrion	81.00	63.82	12.06	4.02	20.10
*PatCASPL4B5*	Chloroplast/Cytoplasm/Golgi apparatus/Nucleus/Vacuole	73.42/51.00/49.00/17.00/49.00	59.30	4.02	4.02	32.66
*PatCASP6*	Cell membrane/Golgi apparatus/Nucleus	54.70/14.00/17.00	49.51	20.59	4.41	25.49
*PatCASPL2B8*	Peroxisome	86.61	62.31	12.56	4.52	20.60
*PatCASPL1A9*	Cell membrane	53.00	46.70	21.43	1.65	30.22
*PatCASPL5B6*	Cell membrane	73.97	59.09	12.99	5.19	22.73
*PatCASPL4D3*	Cell membrane/Golgi apparatus	100.00/69.00	45.66	24.28	5.20	24.86
*PatCASPL4D1*	Cell membrane/Golgi apparatus	99.90/15.00	44.71	22.94	5.29	27.06
*PatCASPL4D6*	Cell membrane/Golgi apparatus	100.00/36.00	51.81	18.07	3.01	27.11
*PatCASPL1A11*	Cell membrane	83.78	32.23	26.45	7.44	33.88
*PatCASP9*	Cell membrane	99.87	46.71	22.75	5.39	25.15
*PatCASPL5B9*	Cell membrane	84.07	64.94	8.44	4.55	22.08
*PatCASPL4B6*	Cell membrane	72.47	56.67	10.00	5.33	28.00
*PatCASPL5A1*	Cell membrane	60.23	48.30	14.20	5.11	32.39
*PatCASPL5A5*	Cell membrane	81.40	60.26	11.92	4.64	23.18
*PatCASP3*	Cell membrane/Nucleus	75.84/49.00	52.40	16.83	2.40	28.37
*PatCASPL5B10*	Cell membrane	84.07	64.94	8.44	4.55	22.08
*PatCASPL4B2*	Cell membrane	73.42	57.36	5.08	2.03	35.53
*PatCASPL5A2*	Cell membrane	60.23	48.30	14.2	5.11	32.39
*PatCASPL5A6*	Cell membrane/Golgi apparatus/Nucleus	81.40/28.00/26.00	49.17	10.50	3.31	37.02
*PatCASP2*	Cell membrane	69.91	50.00	16.35	3.85	29.81
*PatCASPL1D5*	Cell membrane	89.56	39.10	24.36	3.21	33.33
*PatCASPL1D7*	Cell membrane/Nucleus	81.02/36.00	58.55	10.88	4.66	25.91
*PatCASPL4A2*	Chloroplast/Nucleus	62.04/99.00	24.56	16.37	6.43	52.63
*PatCASPL1A7*	Cell membrane/Chloroplast	84.37/36.00	55.61	15.51	3.21	25.67
*PatCASPL1A2*	Cell membrane	88.87	51.06	18.09	1.60	29.26
*PatCASPL5C6*	Cell membrane	96.17	60.53	11.18	1.32	26.97
*PatCASPL1C2*	Cell membrane	100.00	61.11	16.05	3.09	19.75
*PatCASPL4A1*	Chloroplast/Nucleus	62.04/28.00	22.09	20.60	7.16	50.15
*PatCASPL1A6*	Chloroplast	90.17	54.84	17.74	4.30	23.12
*PatCASPL1A1*	Cell membrane	88.87	55.32	17.55	1.60	25.53
*PatCASPL5C8*	Cell membrane	96.17	63.58	10.60	3.31	22.52
*PatCASPL1C1*	Cell membrane	100.00	57.41	19.14	3.09	20.37
*PatCASPL5B3*	Chloroplast	86.47	63.64	8.44	4.55	23.38
*PatCASPL1F4*	Cell membrane/Nucleus	44.19/49.00	46.60	19.37	2.62	31.41
*PatCASPL5B1*	Cell membrane/Chloroplast	86.47/26.00	66.23	9.74	4.55	19.48
*PatCASPL1F1*	Cell membrane	58.9	47.64	20.94	3.14	28.27
*PatCASPL4A10*	Chloroplast/Nucleus	75.73/51.00	30.00	13.06	6.94	50.00
*PatCASPL4A12*	Nucleus	75.56	31.77	12.15	6.35	49.72
*PatCASPL4A13*	Nucleus	75.56	31.77	12.15	6.35	49.72
*PatCASPL1E1*	Cell membrane	51.39	51.27	16.24	6.09	26.40
*PatCASPL1D2*	Cell membrane/Nucleus	79.86/41.00	51.50	12.00	1.50	35.00
*PatCASPL1E3*	Cell membrane	51.39	53.03	16.67	5.56	24.75
*PatCASPL1D3*	Cell membrane/Nucleus	79.86/36.00	47.50	14.00	3.50	35.00
*PatCASPL2C2*	Cell membrane	99.98	67.61	11.36	3.41	17.61
*PatCASPL2C3*	Cell membrane/Chloroplast	99.98/15.00	69.66	12.36	5.62	12.36
*PatCASPL5C1*	Cell membrane	74.95	52.30	17.24	3.45	27.01
*PatCASPL5C4*	Cell membrane	82.53	44.77	18.41	5.44	31.38
*PatCASPL1C7*	Cell membrane	100.00	56.44	16.56	4.29	22.70
*PatCASPL2B4*	Chloroplast/Peroxisome	49.45/81.00	62.38	15.84	3.96	17.82
*PatCASPL4D9*	Cell membrane	50.31	52.02	16.76	10.40	20.81
*PatCASPL4A14*	Cell membrane/Nucleus	79.84/68.00	52.02	16.76	10.40	20.81
*PatCASPL1C5*	Cell membrane	100.00	60.74	15.34	3.68	20.25
*PatCASPL2B3*	Chloroplast/Peroxisome	49.45/81.00	61.39	15.35	3.96	19.31
*PatCASPL4D12*	Nucleus	98.88	53.95	18.86	3.95	23.25
*PatCASPL4A16*	Nucleus	82.80	49.52	12.02	3.37	35.10
*PatCASPL3A2*	Nucleus	69.52	51.20	8.61	4.78	35.41
*PatCASPL4A8*	Nucleus	63.35	32.60	13.70	5.75	47.95
*PatCASPL4D8*	Chloroplast	99.97	49.32	22.30	5.41	22.97
*PatCASPL1F8*	Cell membrane	47.85/95.00	47.42	24.41	5.16	23.00
*PatCASPL1B2*	Chloroplast	59.66	47.26	16.92	6.97	28.86
*PatCASPL2D2*	Cell membrane	98.19	56.67	17.78	2.78	22.78
*PatCASPL3A1*	Nucleus	71.69	50.47	8.49	4.72	36.32
*PatCASPL4A5*	Nucleus	54.68	29.89	13.13	6.15	50.84
*PatCASPL1F5*	Cell membrane	94.16	55.19	18.03	2.19	24.59
*PatCASPL1B4*	Chloroplast/Nucleus	59.66/41.00	49.75	17.41	6.97	25.87
*PatCASPL2D4*	Cell membrane	98.97	60.00	16.11	1.67	22.22
*PatCASPL5A11*	Nucleus	70.61	30.85	16.49	2.66	50.00
*PatCASPL1F11*	Cell membrane	75.84	61.05	12.21	3.49	23.26
*PatCASPL5A10*	Cell membrane/Golgi apparatus/Nucleus	62.28/95.00/17.00	51.12	10.67	3.93	34.27
*PatCASPL1F10*	Cell membrane	55.17	59.30	13.37	3.49	23.84
*PatCASPL4D14*	Cell membrane	94.08	36.19	20.95	4.76	38.10
*PatCASPL2D7*	Cell membrane	98.50	57.38	19.13	2.73	20.77
*PatCASPL2A4*	Cell membrane/Nucleus	98.15	57.37	17.37	3.16	22.11
*PatCASPL2D5*	Cell membrane/Chloroplast/Nucleus	98.5/28.00/26.00	58.47	19.67	3.83	18.03
*PatCASPL2A1*	Cell membrane/Nucleus	98.15/26.00	53.44	53.44	1.06	27.51
*PatCASPL4B6*	Cell membrane/Chloroplast/Cytoplasm/Mitochondrion/Nucleus/Peroxisome	78.42/39.00/49.00/7.00/95.00/17.00	61.11	5.05	3.54	30.30
*PatCASP8*	Cell membrane/Golgi apparatus/Nucleus	54.66/49.00/17.00	51.47	19.12	3.92	25.49
*PatCASPL2B5*	Peroxisome	64.55	63.32	14.07	5.03	17.59
*PatCASPL4B7*	Nucleus	73.42	60.80	4.02	3.02	32.16
*PatCASP5*	Cell membrane/Golgi apparatus/Nucleus	54.66/17.00/95.00	45.59	22.06	5.88	26.47
*PatCASPL2B7*	Peroxisome	49.45	61.31	15.08	5.03	18.59
*PatCASPL1A10*	Cell membrane	66.20	48.90	18.13	3.85	29.12
*PatCASPL5B7*	Cell membrane	73.97	61.04	12.99	4.55	21.43
*PatCASPL4D4*	Cell membrane/Golgi apparatus	100.00/69.00	44.51	24.28	5.20	26.01
*PatCASPL4D2*	Cell membrane/Golgi apparatus	99.99/15.00	40.00	25.88	4.71	29.41
*PatCASPL4D5*	Cell membrane/Golgi apparatus	99.98/36.00	53.61	19.88	3.61	22.89
*PatCASPL1A12*	Cell membrane	98.29	38.51	20.69	6.90	33.91
*PatCASP11*	Cell membrane	99.53	47.52	17.73	7.09	27.66
*PatCASPL5B11*	Cell membrane	84.07	64.94	8.44	4.55	22.08
*PatCASPL4B3*	Cell membrane/Cell wall/Chloroplast/Nucleus/Peroxisome/Vacuole	68.02/16.00/17.00/2.00/95.00/19.00	71.32	3.88	4.65	20.16
*PatCASPL5A3*	Cell membrane	87.02	50.57	13.64	5.11	30.68
*PatCASPL5A7*	Cell membrane/Golgi apparatus/Nucleus	49.05/26.00/28.00	53.37	16.83	2.88	26.92
*PatCASP1*	Cell membrane	66.57	50.83	11.60	4.42	33.15
*PatCASP10*	Cell membrane	99.87	63.12	9.22	5.67	21.99
*PatCASPL5B8*	Cell membrane	84.07	46.71	22.75	5.39	25.15
*PatCASPL4B1*	Cell membrane	97.61	52.27	11.36	7.39	28.98
*PatCASPL5A4*	Cell membrane	67.27	60.90	6.77	9.02	23.31
*PatCASPL5A8*	Cell membrane/Golgi apparatus/Nucleus	49.05/26.00/28.00	50.83	9.94	4.42	34.81
*PatCASP4*	Cell membrane	69.91	48.56	16.83	3.37	31.25
*PatCASPL1D8*	Cell membrane	50.31	56.99	8.81	3.63	30.57
*PatCASPL1D6*	Cell membrane	67.17	41.58	15.79	10.00	32.63
*PatCASPL4A4*	Chloroplast/Nucleus	62.04	22.22	20.47	6.14	51.17
*PatCASPL1A5*	Cell membrane/Chloroplast/Golgi apparatus	90.17/36.00/26.00	57.75	11.76	2.67	27.81
*PatCASPL1A4*	Cell membrane	88.87	52.66	15.96	4.26	27.13
*PatCASPL5C5*	Cell membrane	96.68	42.16	25.49	9.80	22.55
*PatCASPL1C3*	Cell membrane	99.98	58.64	16.67	3.09	21.60
*PatCASPL4A3*	Nucleus	71.28	29.28	13.04	3.77	53.91
*PatCASPL1A8*	Chloroplast	84.37	53.89	17.78	3.89	24.44
*PatCASPL1A3*	Cell membrane	88.87	60.64	13.83	3.19	22.34
*PatCASPL5C7*	Cell membrane	96.17	62.25	10.60	1.32	25.83
*PatCASPL1C4*	Cell membrane	99.51	60.49	15.43	2.47	21.60
*PatCASPL5B2*	Cell membrane/Chloroplast	86.47/28.00	66.23	9.74	4.55	19.48
*PatCASPL1F3*	Cell membrane/Chloroplast/Nucleus	63.75/22.00/27.00	49.74	19.90	5.24	25.13
*PatCASPL5B4*	Chloroplast	52.62	67.53	9.09	5.19	18.18
*PatCASPL1F2*	Cell membrane/Chloroplast	75.73/51.00	51.83	17.28	3.14	27.75
*PatCASPL4A9*	Nucleus	75.56	35.48	9.35	2.58	52.58
*PatCASPL4A11*	Nucleus	51.39	33.33	8.61	4.72	53.33
*PatCASPL1E2*	Cell membrane	79.86	47.21	19.29	5.08	28.43
*PatCASPL1D1*	Cell membrane/Nucleus	79.86/41.00	48.00	13.50	4.00	34.50
*PatCASPL1D4*	Cell membrane/Nucleus	79.86/15.00	50.00	12.00	5.00	33.00
*PatCASPL2C1*	Cell membrane/Chloroplast	99.96/53.00	70.45	11.36	3.41	14.77

**Table 3:** Gene number per chromosome of *CASPL* gene family proteins in *P. cablin*.

Chromosome	Gene Number	Gene Name
A01	1	*PatCASPL5C2*
A02	1	*PatCASPL5C3*
A03	5	*PatCASPL1C8/PatCASPL2B2/PatCASPL4D10/PatCASPL4D13/PatCASPL4A15*
A04	4	*PatCASPL1C6/PatCASPL2B1/PatCASPL4D11/PatCASPL4A17*
A05	6	*PatCASPL3A3/PatCASPL4A7/PatCASPL4D7/PatCASPL1F6/PatCASPL1B1/PatCASPL2D1*
A06	5	*PatCASPL3A4/PatCASPL4A6/PatCASPL1F7/PatCASPL1B3/PatCASPL2D3*
A07	2	*PatCASPL5A12/PatCASPL1F12*
A08	2	*PatCASPL5A9/PatCASPL1F9*
A09	0	
A10	1	*PatCASPL5B5*
A11	3	*PatCASPL1C9/PatCASPL2D8/PatCASPL2A2*
A12	2	*PatCASPL2D6/PatCASPL2A3*
A13	3	*PatCASPL4B8/PatCASP7/PatCASPL2B6*
A14	3	*PatCASPL4B5/PatCASP7/PatCASPL2B6*
A15	5	*PatCASPL1A9/PatCASPL5B6/PatCASPL4D3/PatCASPL4D1/PatCASPL4D6*
A16	1	*PatCASPL1A11*
A17	5	*PatCASP9/PatCASPL5B9/PatCASPL5A1/PatCASPL5A5/PatCASP3*
A18	5	*PatCASPL5B10/PatCASPL4B2/PatCASPL5A2/PatCASPL5A6/PatCASP2*
A19	1	*PatCASPL1D5*
A20	1	*PatCASPL1D7*
A21	5	*PatCASPL4A2/PatCASPL1A7/PatCASPL1A2/PatCASPL5C6/PatCASPL1C2*
A22	5	*PatCASPL4A1/PatCASPL1A6/PatCASPL1A1/PatCASPL5C8/PatCASPL1C1*
A23	2	*PatCASPL5B3/PatCASPL1F4*
A24	2	*PatCASPL5B1/PatCASPL1F1*
A25	1	*PatCASPL4A10*
A26	2	*PatCASPL4A12/PatCASPL4A13*
A27	0	
A28	0	
A29	2	*PatCASPL1E1/PatCASPL1D2*
A30	2	*PatCASPL1E3/PatCASPL1D3*
A31	1	*PatCASPL2C2*
A32	1	*PatCASPL2C3*
A33	1	*PatCASPL5C1*
A34	1	*PatCASPL5C4*
A35	4	*PatCASPL1C7/PatCASPL2B4/PatCASPL4D9/PatCASPL4A14*
A36	4	*PatCASPL1C5/PatCASPL2B3/PatCASPL4D12/PatCASPL4A16*
A37	6	*PatCASPL3A2/PatCASPL4A8/PatCASPL4D8/PatCASPL1F8/PatCASPL1B2/PatCASPL2D2*
A38	5	*PatCASPL3A1/PatCASPL4A5/PatCASPL1F5/PatCASPL1B4/PatCASPL2D4*
A39	2	*PatCASPL5A11/PatCASPL1F11*
A40	2	*PatCASPL5A10/PatCASPL1F10*
A41	0	
A42	1	*PatCASPL4D14*
A43	2	*PatCASPL2D7/PatCASPL2A4*
A44	2	*PatCASPL2D5/PatCASPL2A1*
A45	3	*PatCASPL4B6/PatCASP8/PatCASPL2B5*
A46	3	*PatCASPL4B7/PatCASP5/PatCASPL2B7*
A47	5	*PatCASPL1A10/PatCASPL5B7/PatCASPL4D4/PatCASPL4D2/PatCASPL4D5*
A48	1	*PatCASPL1A12*
A49	6	*PatCASP11/PatCASPL5B11/PatCASPL4B3/PatCASPL5A3/PatCASPL5A7/PatCASP1*
A50	6	*PatCASP10/PatCASPL5B8/PatCASPL4B1/PatCASPL5A4/PatCASPL5A8/PatCASP4*
A51	1	*PatCASPL1D8*
A52	1	*PatCASPL1D6*
A53	5	*PatCASPL4A4/PatCASPL1A5/PatCASPL1A4/PatCASPL5C5/PatCASPL1C3*
A54	5	*PatCASPL4A3/PatCASPL1A8/PatCASPL1A3/PatCASPL5C7/PatCASPL1C4*
A55	2	*PatCASPL5B2/PatCASPL1F3*
A56	2	*PatCASPL5B4/PatCASPL1F2*
A57	1	*PatCASPL4A9*
A58	1	*PatCASPL4A11*
A59	0	
A60	0	
A61	2	*PatCASPL1E2/PatCASPL1D1*
A62	1	*PatCASPL1D4*
A63	1	*PatCASPL2C1*

## References

[1] He Y., Peng F., Deng C., Xiong L., Huang Z., Zhang R., Liu M., Peng C. (2018). Building an octaploid genome and transcriptome of the medicinal plant *Pogostemon cablin* from Lamiales. Sci. Data.

[2] Swamy M.K., Sinniah U.R. (2016). Patchouli (*Pogostemon cablin* Benth.): Botany, agrotechnology and biotechnological aspects. Ind. Crop. Prod..

[3] Wu Y.G., Guo Q.S., He J.C., Lin Y.F., Luo L.J., Liu G.D. (2010). Genetic diversity analysis among and within populations of *Pogostemon cablin* from China with ISSR and SRAP markers. Biochem. Syst. Ecol..

[4] Xie B., Wu X.F., Luo H.T., Huang X.L., Huang F., Zhang Q.Y., Zhou X., Wu H.Q. (2022). Chemical profiling and quality evaluation of *Pogostemon cablin* Benth by liquid chromatography tandem mass spectrometry combined with multivariate statistical analysis. J. Pharm. Biomed. Anal..

[5] Uh Y.R., Jang C.S. (2023). Establishing DNA markers to differentiate agastache rugosa and *Pogostemon cablin*, which are confusedly used as medicinal herbs, using real-time PCR. Food Sci. Biotechnol..

[6] Yan W., Ye Z., Cao S., Yao G., Yu J., Yang D., Chen P., Zhang J., Wu Y. (2021). Transcriptome analysis of two *Pogostemon cablin* chemotypes reveals genes related to patchouli alcohol biosynthesis. PeerJ.

[7] Swamy M., Sinniah U. (2015). A comprehensive review on the phytochemical constituents and pharmacological activities of *Pogostemon cablin* Benth.: An aromatic medicinal plant of industrial importance. Molecules.

[8] Yoon S.C., Je I.G., Cui X., Park H.R., Khang D., Park J.S., Kim S.H., Shin T.Y. (2016). Anti-allergic and anti-inflammatory effects of aqueous extract of *Pogostemon cablin*. Int. J. Mol. Med..

[9] Yan H.J., Xiong Y., Zhang H.Y., He M.L. (2016). In vitro induction and morphological characteristics of octoploid plants in *Pogostemon cablin*. Breed. Sci..

[10] Yan W., Liu X., Cao S., Yu J., Zhang J., Yao G., Yang H., Yang D., Wu Y. (2023). Molecular basis of *Pogostemon cablin* responding to continuous cropping obstacles revealed by integrated transcriptomic, miRNA and metabolomic analyses. Ind. Crop. Prod..

[11] Yan W., Cao S., Wu Y., Ye Z., Zhang C., Yao G., Yu J., Yang D., Zhang J. (2022). Integrated analysis of physiological, mRNA sequencing, and miRNA sequencing data reveals a specific mechanism for the response to continuous cropping obstacles in *Pogostemon cablin* roots. Front. Plant Sci..

[12] Zhou X., Li C., Liu L., Zhao J., Zhang J., Cai Z., Huang X. (2019). Control of fusarium wilt of *Lisianthus* by reassembling the microbial community in infested soil through reductive soil disinfestation. Microbiol. Res..

[13] Dong L., Xu J., Zhang L., Cheng R., Wei G., Su H., Yang J., Qian J., Xu R., Chen S. (2018). Rhizospheric microbial communities are driven by *Panax ginseng* at different growth stages and biocontrol bacteria alleviates replanting mortality. Acta Pharm. Sin. B.

[14] Zhao Y., Qin X., Tian X., Yang T., Deng R., Huang J. (2021). Effects of continuous cropping of *Pinellia Ternata* (Thunb.) Breit. on soil physicochemical properties, enzyme activities, microbial communities and functional genes. Chem. Biol. Technol. Agric..

[15] Xu Y., Wu Y.G., Chen Y., Zhang J.F., Song X.Q., Zhu G.P., Hu X.W. (2015). Autotoxicity in *Pogostemon cablin* and their allelochemicals. Rev. Bras. Farmacogn..

[16] Yan W., Cao S., Liu X., Yao G., Yu J., Zhang J., Bian T., Yu W., Wu Y. (2022). Combined physiological and transcriptome analysis revealed the response mechanism of *Pogostemon cablin* roots to p-Hydroxybenzoic Acid. Front. Plant Sci..

[17] Geldner N. (2013). Casparian strips. Curr. Biol..

[18] Barbosa I.C.R., Rojas-Murcia N., Geldner N. (2019). The Casparian strip—One ring to bring cell biology to lignification?. Curr. Opin. Biotechnol..

[19] De Bang T.C., Lay K.S., Scheible W.R., Takahashi H. (2017). Small peptide signaling pathways modulating macronutrient utilization in plants. Curr. Opin. Plant Biol..

[20] Rojas-Murcia N., Hématy K., Lee Y., Emonet A., Ursache R., Fujita S., De Bellis D., Geldner N. (2020). High-order mutants reveal an essential requirement for peroxidases but not laccases in Casparian strip lignification. Proc. Natl. Acad. Sci. USA.

[21] Roppolo D., Boeckmann B., Pfister A., Boutet E., Rubio M.C., Dénervaud-Tendon V., Vermeer J.E.M., Gheyselinck J., Xenarios I., Geldner N. (2014). Functional and evolutionary analysis of the CASPARIAN STRIP MEMBRANE DOMAIN PROTEIN Family. Plant Physiol..

[22] Pfister A., Barberon M., Alassimone J., Kalmbach L., Lee Y., Vermeer J.E., Yamazaki M., Li G., Maurel C., Takano J. (2014). A Receptor-like kinase kutant with absent endodermal diffusion barrier displays selective nutrient homeostasis defects. eLife.

[23] Hosmani P.S., Kamiya T., Danku J., Naseer S., Geldner N., Guerinot M.L., Salt D.E. (2013). Dirigent domain-containing protein is part of the machinery required for formation of the lignin-based Casparian strip in the root. Proc. Natl. Acad. Sci. USA.

[24] Kamiya T., Borghi M., Wang P., Danku J.M.C., Kalmbach L., Hosmani P.S., Naseer S., Fujiwara T., Geldner N., Salt D.E. (2015). The MYB36 transcription factor orchestrates Casparian strip formation. Proc. Natl. Acad. Sci. USA.

[25] Fujita S. (2021). CASPARIAN STRIP INTEGRITY FACTOR (CIF) family peptides—Regulator of plant extracellular barriers. Peptides.

[26] Kim Y.Y., Kim D.Y., Shim D., Song W.Y., Lee J., Schroeder J.I., Kim S., Moran N., Lee Y. (2008). Expression of the novel wheat gene TM20 confers enhanced cadmium tolerance to bakers’ yeast. J. Biol. Chem..

[27] Barbosa I.C.R., De Bellis D., Flückiger I., Bellani E., Grangé-Guerment M., Hématy K., Geldner N. (2023). Directed growth and fusion of membrane-wall microdomains requires CASP-mediated inhibition and displacement of secretory foci. Nat. Commun..

[28] Roppolo D., De Rybel B., Tendon V.D., Pfister A., Alassimone J., Vermeer J.E.M., Yamazaki M., Stierhof Y.-D., Beeckman T., Geldner N. (2011). A novel protein family mediates Casparian strip formation in the endodermis. Nature.

[29] Wang X., Zhang Y., Wang L., Pan Z., He S., Gao Q., Chen B., Gong W., Du X. (2020). Casparian strip membrane domain proteins in *Gossypium Arboreum*: Genome-wide identification and negative regulation of lateral root growth. BMC Genom..

[30] Ji Y., Zhang C., Li D., Lai Z. (2021). Genome-wide identification and analysis of CASP gene family in banana. Subtrop. Agric. Res..

[31] Chen T., Liao X. (2022). Identification and analysis of whole genome of *Litchi* CASP family. Mol. Plant Breed..

[32] Liu L., Wei X., Yang Z., Yuan F., Han G., Guo J., Wang B. (2023). SbCASP-LP1C1 improves salt exclusion by enhancing the root apoplastic barrier. Plant Mol. Biol..

[33] Chen F., Fang P., Peng Y., Zeng W., Zhao X., Ding Y., Zhuang Z., Gao Q., Ren B. (2019). comparative proteomics of salt-tolerant and salt-sensitive Maize inbred lines to reveal the molecular mechanism of salt tolerance. Int. J. Mol. Sci..

[34] Chen T., Cai X., Wu X., Karahara I., Schreiber L., Lin J. (2011). Casparian strip development and its potential function in salt tolerance. Plant Signal. Behav..

[35] Yang J., Ding C., Xu B., Chen C., Narsai R., Whelan J., Hu Z., Zhang M. (2015). A Casparian strip domain-like gene, *CASPL*, negatively alters growth and cold tolerance. Sci. Rep..

[36] Lee M., Jeon H.S., Kim S.H., Chung J.H., Roppolo D., Lee H., Cho H.J., Tobimatsu Y., Ralph J., Park O.K. (2019). Lignin-based barrier restricts pathogens to the infection site and confers resistance in plants. EMBO J..

[37] McGinnis S., Madden T.L. (2004). BLAST: At the core of a powerful and diverse set of sequence analysis tools. Nucleic Acids Res..

[38] Eddy S.R. (2011). Accelerated profile HMM searches. PLoS Comput. Biol..

[39] Gasteiger E. (2003). ExPASy: The proteomics server for in-depth protein knowledge and analysis. Nucleic Acids Res..

[40] Geourjon C., Deléage G. (1995). SOPMA: Significant improvements in protein secondary structure prediction by Consensus prediction from multiple alignments. Bioinformatics.

[41] Bailey T.L., Elkan C. (1994). Fitting a mixture model by expectation maximization to discover motifs in biopolymers. Proc. Int. Conf. Intell. Syst. Mol. Biol..

[42] Lu S., Wang J., Chitsaz F., Derbyshire M.K., Geer R.C., Gonzales N.R., Gwadz M., Hurwitz D.I., Marchler G.H., Song J.S. (2020). CDD/SPARCLE: The conserved domain database in 2020. Nucleic Acids Res..

[43] Tamura K., Stecher G., Kumar S. (2021). MEGA11: Molecular evolutionary genetics analysis version 11. Mol. Biol. Evol..

[44] Lescot M. (2002). PlantCARE, a database of plant cis-acting regulatory elements and a portal to tools for in silico analysis of promoter sequences. Nucleic Acids Res..

[45] Tian R., Yang Y., Chen M. (2020). Genome-wide survey of the amino acid transporter gene family in wheat (*Triticum Aestivum L.*): Identification, expression analysis and response to abiotic stress. J. Biol. Macromol..

[46] Piegu B., Guyot R., Picault N., Roulin A., Sanyal A., Kim H., Collura K., Brar D.S., Jackson S., Wing R.A. (2006). Doubling genome size without polyploidization: Dynamics of retrotransposition-driven genomic expansions in *Oryza australiensis*, a wild relative of rice. Genome Res..

[47] Zhao M., Ma J. (2013). Co-evolution of plant LTR-retrotransposons and their host genomes. Protein Cell.

[48] Shen Y., Li W., Zeng Y., Li Z., Chen Y., Zhang J., Zhao H., Feng L., Ma D., Mo X. (2022). Chromosome-level and haplotype-resolved genome provides insight into the tetraploid hybrid origin of patchouli. Nat. Commun..

[49] Fei Z. (2021). Analysis and Functional Identification of CASP Family Members in Wheat.

[50] Rushil M. (2019). Cloning and in silico analysis of Casparian strip membrane domain protein (CASP) from rice. Madras Agric. J..

[51] Zhang W., Sun P., He Q., Shu F., Deng H. (2018). Transcriptome analysis of near-isogenic line provides novel insights into genes associated with panicle traits regulation in rice. PLoS ONE.

[52] Zhang J., He L., Wu Y., Ma W., Chen H., Ye Z. (2018). Comparative proteomic analysis of *Pogostemon cablin* leaves after continuous cropping. Protein Expr. Purif..

[53] Zeng J., Liu J., Lu C., Ou X., Luo K., Li C., He M., Zhang H., Yan H. (2020). Intercropping with turmeric or ginger reduce the continuous cropping obstacles that affect *Pogostemon cablin* (patchouli). Front. Microbiol..

[54] Liu Q., Li K., Guo X., Ma L., Guo Y., Liu Z. (2019). Developmental characteristics of grapevine seedlings root border cells and their response to p-Hydroxybenzoic acid. Plant Soil.

[55] Tan G., Liu Y., Peng S., Yin H., Meng D., Tao J., Gu Y., Li J., Yang S., Xiao N. (2021). Soil potentials to resist continuous cropping obstacle: Three field cases. Environ. Res..

[56] Chen Y., Du J., Li Y., Tang H., Yin Z., Yang L., Ding X. (2022). Evolutions and managements of soil microbial community structure drove by continuous cropping. Front. Microbiol..

[57] Liu S., Wang Z., Niu J., Dang K., Zhang S., Wang S., Wang Z. (2021). Changes in physicochemical properties, enzymatic activities, and the microbial community of soil significantly influence the continuous cropping of *Panax quinquefolius* L. (American Ginseng). Plant Soil.

[58] Zeeshan Ul Haq M., Yu J., Yao G., Yang H., Iqbal H.A., Tahir H., Cui H., Liu Y., Wu Y. (2023). A systematic review on the continuous cropping obstacles and control strategies in medicinal plants. Int. J. Mol. Sci..

[59] Chen C., Chen H., Zhang Y., Thomas H.R., Frank M.H., He Y., Xia R. (2020). TBtools: An integrative toolkit developed for interactive analyses of big biological data. Mol. Plant..

[60] Camacho C., Coulouris G., Avagyan V., Ma N., Papadopoulos J., Bealer K., Madden T.L. (2009). BLAST^+^: Architecture and applications. BMC Bioinform..

[61] Paysan-Lafosse T., Blum M., Chuguransky S., Grego T., Pinto B.L., Salazar G.A., Bileschi M.L., Bork P., Bridge A., Colwell L. (2023). InterPro in 2022. Nucleic Acids Res..

[62] Potter S.C., Luciani A., Eddy S.R., Park Y., Lopez R., Finn R.D. (2018). HMMER web server: 2018 update. Nucleic Acids Res..

[63] Artimo P., Jonnalagedda M., Arnold K., Baratin D., Sardi G., De Castro E., Duvaud S., Flegel V., Fortier A., Gasteiger E. (2012). ExPASy: SIB bioinformatics resource portal. Nucleic Acids Res..

[64] Chou K.C., Shen H.B. (2010). Plant-mPLoc: A Top-down strategy to augment the power for predicting plant protein subcellular localization. PLoS ONE.

[65] Xiong E., Zheng C., Wu X., Wang W. (2016). Protein subcellular location: The gap between prediction and experimentation. Plant Mol. Biol. Rep..

[66] Bailey T.L., Boden M., Buske F.A., Frith M., Grant C.E., Clementi L., Ren J., Li W.W., Noble W.S. (2009). MEME SUITE: Tools for motif discovery and searching. Nucleic Acids Res..

[67] Briesemeister S., Rahnenführer J., Kohlbacher O. (2010). YLoc—An interpretable web server for predicting subcellular localization. Nucleic Acids Res..

[68] Mistry J., Chuguransky S., Williams L., Qureshi M., Salazar G.A., Sonnhammer E.L.L., Tosatto S.C.E., Paladin L., Raj S., Richardson L.J. (2021). Pfam: The protein families database in 2021. Nucleic Acids Res..

[69] Edgar R.C. (2004). MUSCLE: Multiple sequence alignment with high accuracy and high throughput. Nucleic Acids Res..

[70] Darriba D., Taboada G.L., Doallo R., Posada D. (2011). ProtTest 3: Fast selection of best-fit models of protein evolution. Bioinformatics.

[71] Rombauts S., Dehais P., Van Montagu M., Rouze P. (1999). PlantCARE, a plant cis-acting regulatory element database. Nucleic Acids Res..

[72] Zhang H., Deng W., Lu C., He M., Yan H. (2022). SMRT Sequencing of full-length transcriptome and gene expression analysis in two chemical types of *Pogostemon cablin* (Blanco) Benth. PeerJ.

